# Viability of five different pre- and intraoperative imaging methods for autologous breast reconstruction

**DOI:** 10.1007/s10353-016-0449-6

**Published:** 2016-10-19

**Authors:** K. F. Schrögendorfer, S. Nickl, M. Keck, D. B. Lumenta, C. Loewe, M. Gschwandtner, W. Haslik, J. Nedomansky

**Affiliations:** 1 Division of Plastic and Reconstructive Surgery, Department of Surgery, Medical University of Vienna, Währinger Guertel 18–20, 1090 Vienna, Austria; 2 Department of Radiology, Division of Cardiovascular and Interventional Radiology, Medical University of Vienna, Vienna, Austria; 3 Department of Angiology, Medical University of Vienna, Vienna, Austria

**Keywords:** Autologous breast reconstruction, Computed tomography angiography, Laser Doppler imaging, Indocyanine green, Imaging methods

## Abstract

**Background:**

Autologous breast reconstruction is an integral part in the treatment of breast cancer. While computed tomography angiography (CTA) is an established preoperative diagnostic tool for microsurgeons, no study has so far evaluated and compared five different imaging methods and their value for the reconstructive team. In order to determine the feasibility of each of the tools for routine or specialized diagnostic application, the methods’ efficiency and informative value were analyzed.

**Methods:**

We retrospectively analyzed imaging data of 41 patients used for perforator location and assessment for regional perfusion and vessel patency in patients undergoing autologous breast reconstruction with deep inferior epigastric perforator flap (DIEP), transverse rectus abdominis muscle flap (TRAM), or transverse myocutaneous gracilis flap (TMG). Five different imaging techniques were used: hand held Doppler (HHD), CT angiography (CTA), macroscopic indocyanine green (ICG) video angiography, microscope-integrated ICG video angiography, and laser Doppler imaging (LDI).

**Results:**

CTA proved to be the best tool for preoperative determination of the highly variable anatomy of the abdominal region, whereas HHD showed the same information on perforator localization with some false-positive results. Intraoperative HHD was an excellent tool for dissection and vessel patency judgment. Microscope-integrated ICG was an excellent tool to document the patency of microanastomoses. In our series, macroscopic perfusion measurement with ICG or LDI was only justified in special situations, where information on perfusion of abdominal or mastectomy flaps was required. LDI did not add any additional information.

**Conclusion:**

Preoperative assessment should be performed by CTA with verification of the perforator location by HHD. Intraoperative HHD and microscope-integrated ICG contribute most toward the evaluation of vessel patency. ICG and LDI should only be used for special indications.

## Introduction

Breast reconstruction is an integral part in the treatment of breast cancer to overcome the sequelae associated with tumor resection and radiotherapy. Several reconstructive strategies have become available, depending on the requirements for each patient. One of the main objectives is to provide a safe and reliable method to recreate an esthetically pleasing breast. When removal of all breast tissue is necessary, autologous breast reconstruction has proven to deliver optimal results in terms of shape, natural appearance, and pliability [1–5]. However, autologous breast reconstruction with deep inferior epigastric perforator flap (DIEP), transverse rectus abdominis muscle flap (TRAM), or transverse myocutaneous gracilis flap (TMG) is associated with longer operation time, advanced surgical technique involving microsurgery, and an increased risk of flap loss compared with implants or pedicled myocutaneous latissimus dorsi flaps (MLD) [6]. Partial or complete flap loss in this reconstructive setting is always a catastrophe and must be avoided at all costs with all precautions taken. Therefore, many technical devices have been developed to provide the surgeon with information on the localization of perforators, patency of vessels or microanastomoses, and perfusion of tissue with the primary aim of minimizing complications and optimizing outcome. All of these techniques have been described in the literature to varying extent [7–15]. However, the efficiency of different diagnostic methods and their redundant information with respect to each stage in microsurgical breast reconstruction have not been weighed against each other in a summarized context.

One approach to comparing different diagnostic measurements is to analyze each of them based on distinct parameters such as interpretive value, time efficiency, associated risks, availability, and costs.

In general, diagnostic measurements can be differentiated into the three distinct phases of microsurgical breast reconstruction: preoperatively, intraoperatively before microsurgery, and intraoperatively after microsurgery. In order to determine the feasibility of each of the tools for routine or specialized diagnostic application, we analyzed each of the methods’ efficiency and informative value for the surgeon during the aforementioned phases.

## Materials and methods

We retrospectively analyzed imaging data used for perforator location and patency as well as for assessment of regional perfusion and vessel patency in all patients undergoing autologous breast reconstruction with DIEP, TRAM, or TMG in our institution during a 3-year period. This study was approved by the local ethics committee (EK 184/2011).

Five different imaging techniques were used: handheld Doppler (HHD), computed tomography angiography (CTA), macroscopic indocyanine green (ICG) video angiography, microscope-integrated ICG video angiography (mi ICG), and laser Doppler imaging (LDI). These methods were used and applied in all or several of the three different stages of breast reconstruction (Table 1).

**Table 1:** Synopsis of all imaging methods in the different phases of breast reconstruction

Phases	CTA	HHD	ICG macro	ICG micro	LDI
Preoperative	Yes	Yes	No	No	No
Intraoperative before microanastomoses	No	Yes	Yes	No	Yes
Intraoperative after microanastomoses	No	Yes	No	Yes	Yes

*CTA* computed tomography angiography, *HHD* handheld Doppler, *ICG* *macro* macroscopic indocyanine green video angiography, *ICG* *micro* microscope-integrated ICG video angiography, *LDI* laser Doppler imaging

HHD (bidirectional Doppler probe, Multi Dopplex II, Huntleigh Technology Ltd., UK) was used for the detection of the perforator location and the assessment of its flow. Preoperatively, the abdominal donor site was examined in order to localize perforators, and the appropriate sites were marked with a waterproof ink marker. Intraoperatively, HHD was used to confirm the integrity of the dissected perforators before microsurgery, and to judge vessel patency after microsurgery.

CTA (Siemens Somatom Definition Flash using 120 kV and dose adapted mAs) with intravenously applied contrast agent (90 ml of Iomeron® 400 mg/ml, Bracco, Austria; with an injection speed of 5 ml/s followed by a saline flush of 40 ml) was performed preoperatively to assess the vessel, muscle, soft tissue, and intra-abdominal anatomy of the abdominal donor region. For improved visualization, the acquired images were reconstructed using volume-rendered and maximum intensity projection techniques. The number and diameter of perforators were determined. The course of the deep inferior epigastric vessels and their branches in relation to the rectus abdominis muscle as well as related morphology (e.g., rectus diastasis) were evaluated. A transparent grid was used to allow for optimal projection of all detected data in relation to the umbilicus on a data sheet, and was marked on the abdominal donor region with an ink marker on the day before surgery. Intraoperative findings were defined as the standard, and HHD vs. CTA was compared with these findings.

Macroscopic ICG angiography is a near-infrared technique of dynamic laser fluorescence videography (IC-View®, Pulsion Medical Systems, Munich, Germany) [16] for assessment of tissue perfusion. It was only used in cases where large tissue harvests were necessary in order to visualize blood flow and estimate the maximal possible size of the raised abdominal tissue allowing for sufficient flap perfusion. After cleaning the region of interest (ROI) from blood [17], a dose of 0.2 mg/kg ICG was administered intravenously. The perfusion dynamics were visualized in real time and simultaneously stored in the system for subsequent analysis (IC-Calc®; Pulsion Medical Systems, Munich, Germany). The skin perfusion of the raised flap in the ROI was expressed as mean pixel intensity; thereby the site of the perforator, the very edge of the flap, and the later discarded part of the flap were determined as ROIs.

Microscope-integrated ICG video angiography: Microscope-integrated ICG was performed with three different microscopes to determine the patency of the microsurgery, depending on the availability of each device: MÖLLER Hi-R 20–1000G ICG®, ZEISS OPMI Pentero Infrared 800®, and LEICA M720 OH5®. All systems contain a three-chip HD and a TV camera with a near-infrared spectrum. After intravenous application of 0.2 mg/kg ICG, the blood flow in the vessels was visualized and was recorded by an infrared camera and displayed on a monitor adjacent to the microscope allowing for the assessment of vessel patency.

A PIM II LDI device (Laser Doppler Perfusion Imager, Perimed, Sweden) was used in selected cases to evaluate the perfusion of abdominal flaps before and after microsurgery. The ROI was scanned by a laser beam resulting in a color-coded image of the blood perfusion.

The five methods were independently analyzed by two surgeons involved in the patients’ treatment, and the quality of information was assessed using the following parameters:

- Interpretive value (false positive, false negative)
- Handling
- Redundant information
- Time efficiency for the surgeon (presence of patient required for analysis, intraoperative application, effect on overall duration of surgery)
- Time efficiency for the patient (e.g., separate appointments, waiting lists, effect on overall duration of surgery)
- Associated risks (e.g., radiation, extended duration of surgery)
- Availability (e.g., waiting lists, specialized centers etc.)
- Value for money (costs for patient/health-care system and value of information for surgeon)

Each of the parameters was judged against a rating scale, which ranged from excellent to poor, with good and fair representing intermediate evaluations (Tables 2, 3 and 4).

**Table 2:** Detailed synopsis of imaging methods in the preoperative phase

	Preoperative phase	
Imaging method	CTA	*HHD*
Interpretive value	High, displays all relevant details	*No qualitative assessment possible*
Handling	If established, no long waiting list, RTA familiar with procedure	Easy
Redundant information	No	*CTA*
Time efficiency for surgeon	Analysis can be done independent of the presence of patient	*Evaluation can only be done on the patient, 45 min*
Time efficiency for patient	2nd appointment necessary, but quick investigation, and quick marking preoperative	*Long duration of investigation, 45 min*
Associated risks	*Radiation and iv contrast agent*	No
Availability	CTA is available in all hospitals providing breast reconstruction	Unlimited
Value for money	Some costs, but highly effective	*Cheap, but only limited information*

*Normal type* represents “excellent” evaluation on rating scale*; italic type* represents “good” evaluation on rating scale; *CTA* CT angiography; *RTA* radiotechnical assistant

**Table 3:** Detailed synopsis of imaging methods in the intraoperative phase before microanastomoses

	Intraoperative pre-anastomoses		
Imaging method	HHD	*Macroscopic ICG*	**LDI**
Interpretive value	Adequate	Dynamic investigation	*Static investigation*
Handling	Easy	*Requires training*	*Requires training*
Redundant information	No	*LDI*	*ICG*
Time efficiency for surgeon	Quick assessment of perforators	*10 min extension of op time*	***30 min extension of op time***
Time efficiency for patient	No time consuming procedure	*10 min extension of op time*	***30 min extension of operating time***
Associated risks	No	*I.V. application of ICG*	No
Availability	Easy available	*Special equipment*	*Special equipment*
Value for money	Good information, quick evaluation, eases preparation	*Only if clinical judgement is in doubt (selected cases)*	**No additional information in comparison with ICG**

*Normal type* represents “excellent” evaluation on rating scale;* bold type* represents “poor” evaluation on rating scale*; bold and italic type* represents “fair” evaluation on rating scale; *italic type* represents “good” evaluation on rating scale

**Table 4:** Detailed synopsis of imaging methods in the intraoperative phase after microanastomoses

	Post anastomoses	
Imaging method	Microscope integrated ICG	*HHD*
Interpretive value	Excellent display of vessel patency	*No qualitative assessment possible*
Handling	Easy	Easy
Redundant information	No	*ICG micro*
Time efficiency for surgeon	5 min	*Easy, but difficult to interpret (vein)*
Time efficiency for patient	5 min	Quick procedure
Associated risks	*i.v. application of ICG*	No
Availability	**High costs, integrated only in new microscopes **	Easy available
Value for money	Expensive, but best information about patency of microanastomoses	*Cheap, but no reliable information*

*Normal type* represents “excellent” evaluation on rating scale;* bold type* represents “poor” evaluation on rating scale*;* *italic type* represents “good” evaluation on rating scale

### Statistical analysis

For descriptive statistics we used IBM® SPSS® Statistics 18 (Chicago Ill.). Values are expressed as mean and standard deviation for parametric and median (minimum–maximum) for nonparametric data.

## Results

A total of 41 patients with 43 autologous breast reconstructions were treated in our hospital in the study period. Among these patients, 29 DIEP flaps (28 unilateral, 1 bilateral), five unilateral pedicled TRAM flaps, and nine TMG flaps (eight unilateral, one bilateral) were performed; 17 patients underwent immediate reconstructions, and 24 underwent secondary reconstructions. In this collective, 41 HHD, 25 CTA, eight ICG microscopic-based angiographies, five macroscopic flap ICG angiographies, and three LDI methods were performed. We did not observe any total or partial flap loss. There was no occurrence of postoperative hematoma or infection, seroma formation occurred in four patients, and one patient developed skin necrosis of the abdominal donor site after harvest of a DIEP flap.

The results of the imaging methods were stratified into three different time periods: preoperative phase, intraoperative phase pre-microsurgery, and intraoperative phase post-microsurgery. The assessment of each respective imaging method is summarized in Tables 2, 3 and 4.

### Preoperative phase (HHD, CTA)

CTA was found to have a 100 % sensitivity and specificity rate for perforator localization (vs. intraoperatively confirmed anatomy). Rectus diastasis was found in 23 of 25 patients during CTA (median: 2.5 cm, 0–4.5 cm). The mean number of perforators was 4.28 ± 0.99, with an average diameter of 1.73 ± 0.47 mm. The scan time for CTA was 1.5 s; the mean time for image analysis by surgeons was 15 ± 10 min. The transfer of important markings to the patient’s surface took about 5 min.

By contrast, the HHD had 100 % sensitivity and 97.1 % specificity (vs. intraoperatively confirmed anatomy). Owing to the nature of HHD there was no possibility to quantify vessel diameter or to identify the anatomical course through the muscle. The time needed for the surgeon to localize and mark the perforators was 15–45 min depending on the vessel diameter, its course, as well as the patient’s body weight and thickness of the subcutaneous tissue.

### Intraoperative phase (HHD, macroscopic ICG, LDI)

During the intraoperative phase, HHD was repeated under sterile conditions to confirm perforator localization during flap harvest and to evaluate the perforator patency after intramuscular dissection. In this context, HHD was found to be reliable, and no false-positive or false-negative results were noted.

**Fig. 1:**
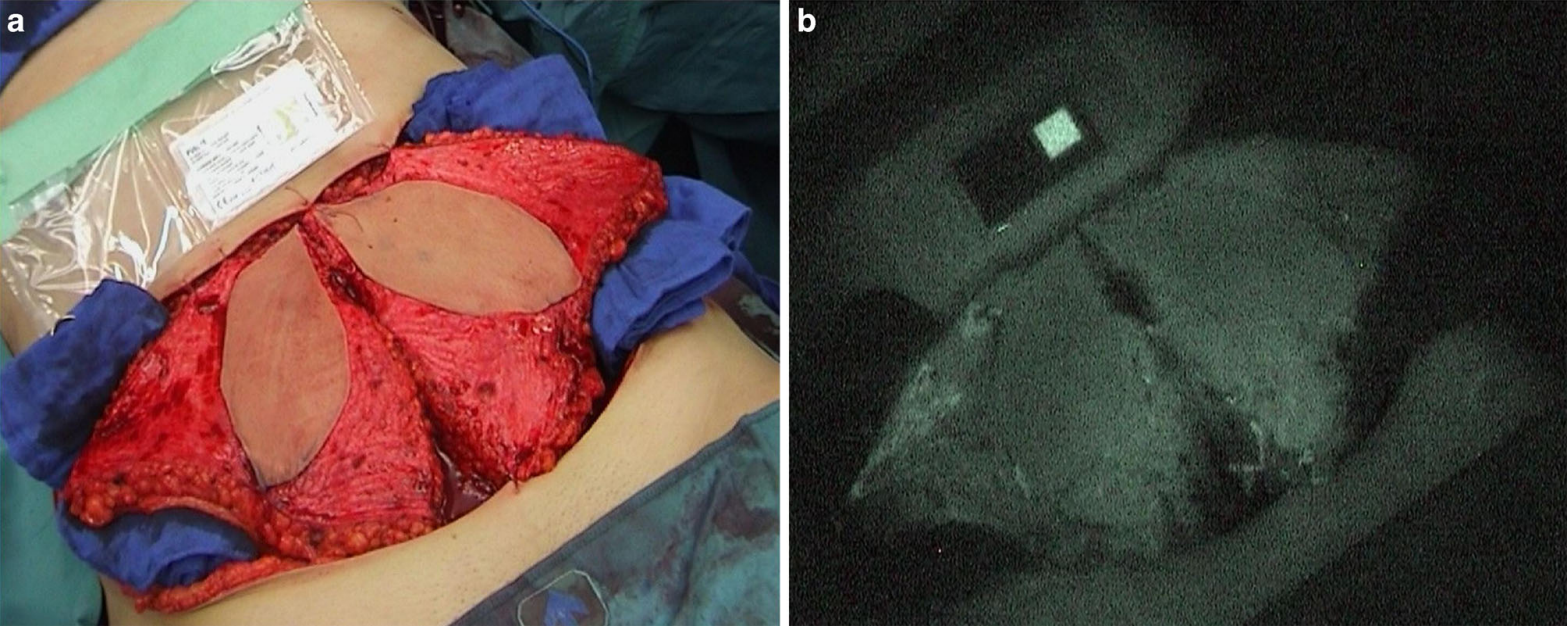
**a** Real-time view of a bilateral DIEP before ICG video. **b** Steady-state ICG view after application of ICG dye. Note homogeneous perfusion of both entire flaps

**Fig. 2:**
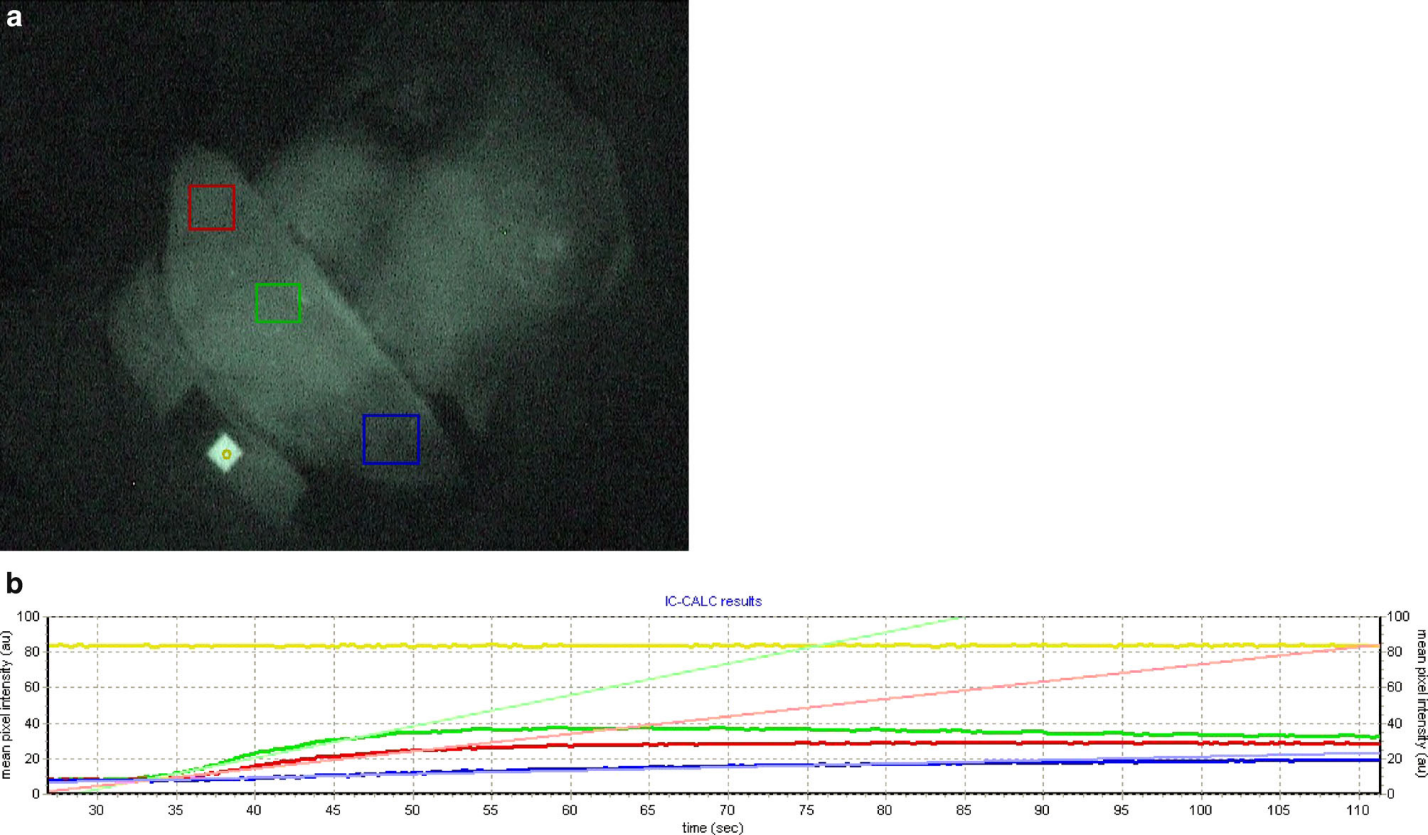
**a** Raised DIEP flap in ICG view, steady state after ICG dye application. *Green area:* localization of perforator; *red area:* border of flap design; *blue area:* discarded area. **b** *Green curve:* site of perforator; *red curve:* edge of flap; *blue curve:* discarded tissue of the flap

The median mean pixel intensity of macroscopic ICG was 39.6 (37.6–44.8) in the area of the perforator, 31.2 (29.1–38.9) at the end of the flaps, and 21.9 (19.4–30.1) in the discarded parts of the flaps (Figs. 1 and 2).

LDI was used in three cases as a noninvasive method for determination of the flap perfusion. In one case, flap ischemia occurred during skin closure, and LDI confirmed compromised flap perfusion before microsurgical revision.

In cases where macroscopic ICG or LDI was used, no malperfused region was detected intraoperatively, and no skin or fat necrosis occurred during postoperative follow-up.

### Post-microsurgery phase (microscope-integrated ICG, HHD)

Microscope-integrated ICG demonstrated vessel patency in six out of eight cases. In the remainder, no flow was shown on ICG, and resulted in the decision to perform immediate microsurgical revision (*n* = 2); HHD was performed in all cases to evaluate Doppler signaling in the distal part of the microanastomosis. Arterial acoustic detection was clearly audible in all patent anastomoses; however, venous signaling was difficult to classify, and did not allow for definitive judgment of the vessel patency.

## Discussion

Autologous free flaps are an essential part of breast reconstruction, albeit technically more demanding and more time consuming than implant-related techniques or pedicled flaps. Nonetheless, their results bear the most advantages of autologous tissue reconstruction: natural feel, no follow-up surgery, and no foreign-body-related reactions.

A key element for a successful autologous breast reconstruction is identifying the entire course of the tissue supplying blood vessels. Accurate preoperative imaging is fundamental when developing a surgical plan in order to refrain from time-consuming intraoperative assessments especially in the presence of multiple perforators of similar caliber and flow [18, 19].

In our retrospective analysis we evaluated imaging techniques whose application provided the surgical team with relevant clinical information during three operative stages. In the preoperative phase, CTA was performed on all patients undergoing abdominal flap reconstruction. CTA has proven to be a method of choice for the preoperative investigation of perforators and has aided in reducing operative time [13, 20]. It allowed for the precise demonstration of their intramuscular course, localization, and caliber. In addition, the radiation dose applied can be minimized by choosing the appropriate ROI, and was the case in our patient collective analyzed in conjunction with previous reports [7, 21]. While examination planning and technical infrastructure require more time in the preoperative phase, the benefits of CTA have proven to offset these shortcomings (see Table 1, Preoperative). So far, magnetic resonance angiography has been evaluated in different studies, but has not yet gained the same popularity as CTA. However, this technique could be a radiation-free option for the future [22–24].

**Fig. 3:**
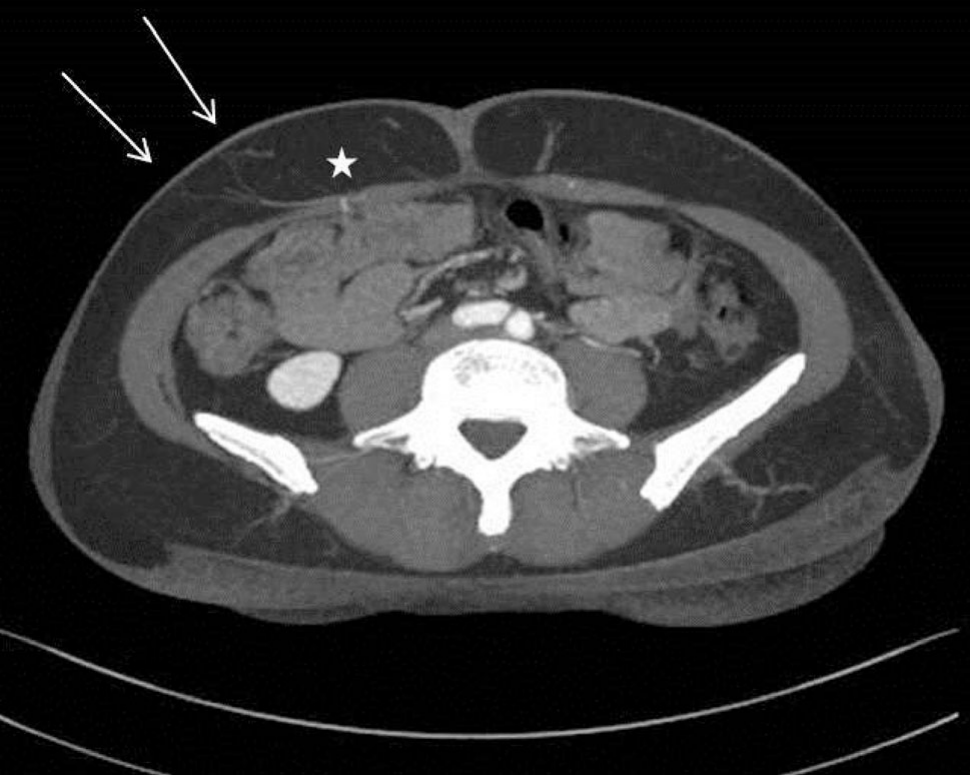
MDCT picture before DIEP breast reconstruction showing differences between actual position of perforator and Doppler signal. *Star* indicates real position of perforator piercing the fascia. *White arrow* indicates the position of audible preoperative Doppler signal

HHD is a noninvasive operator-dependent procedure, which can be applied in a variety of clinical contexts. While its associated costs are limited to its one-time purchase, it does not allow for quantification of blood flow and visualization of anatomical structures. Additionally, it required the surgeon’s and patient’s presence during the entire procedure, and did not permit a post hoc analysis of data in contrast to CTA. From a surgical point of view, it was more convenient to have a preoperative examination performed in a time- and location-independent setting. HHD also delivered false-positive results with regard to perforator location in comparison to CTA (Fig. 3), which was described before [8, 25]. HHD proved to be an excellent device for validating the CTA markings preoperatively.

Intraoperatively, HHD aided in facilitating perforator dissection and evaluating vessel patency, and was hence an essential tool for the operative team.

In our study microscope-integrated ICG video angiography reliably detected vessel patency/occlusion after microsurgery leading to immediate revision. In these cases, neither flap revision was required nor partial or total flap loss was observed. However, whether this was related to the application of this method remains hypothetical in this study’s context. In a report by Holm et al., ICG demonstrated 100 % sensitivity and 86 % specificity for the detection of microvascular thrombosis in 20 patients with clinical signs of flap malperfusion [10]. This objective method allows for the intraoperative evaluation of vessel patency, possibly reduces the requirement for intraoperative empty-and-refill tests, and facilitates immediate documentation of the microsurgical results [14].

The difference between the microscope-integrated and the macroscopic video camera-based ICG system lies essentially in the smaller field of vision through the microscope. A camera-only-based system allows for portability and variable use under different clinical circumstances. It proved to be a reliable method for performing dynamic perfusion investigations in DIEP and SIEA flaps [9]. From our point of view, its use provided an objective intraoperative dynamic evaluation of tissue perfusion, and could, for example, be applied for visualizing perfusion in post-mastectomy skin flaps during immediate breast reconstruction [26], large single perforator flaps, or bilateral DIEP flaps, if viability is in doubt.

The laser-based noninvasive perfusion measurement delivered static information, and did not require intravenous application of a contrast agent as in ICG-based methods. It is therefore an objective method for determining the final steady state of tissue perfusion in a research setting. However, its clinical setup was quite cumbersome, and in fact prolonged the operative procedure without providing more information than the aforementioned method.

## Conclusion

- In our study, CTA proved to be the best tool for preoperative determination of the highly variable anatomy of the abdominal region and should be used in any patient despite cost and radiation dose.
- HHD provided the same information on perforator localization preoperatively, with some false-positive results, but it does not display the course of the vessel. Therefore, this investigation need not be done preoperatively, but can be used for confirmation of CTA markings.
- In the intraoperative setting, HHD is an indispensable, quick, reliable, and cheap tool for dissection and vessel patency judgment with no burden for the patient.
- Macroscopic ICG is only justified in special situations where information on the perfusion border of the abdominal flaps or mastectomy flaps is needed.
- Microscope-integrated ICG is an excellent tool to document the patency of the mircoanastomoses and can also be used to check the viability of the mastectomy flap.
- LDI proved to be a noninvasive tool for the assessment of skin perfusion; however, it does not add additional information to the other techniques and was therefore abandoned.
